# Effects of Intercropping with Potato Onion on the Growth of Tomato and Rhizosphere Alkaline Phosphatase Genes Diversity

**DOI:** 10.3389/fpls.2016.00846

**Published:** 2016-06-15

**Authors:** Xia Wu, Fengzhi Wu, Xingang Zhou, Xuepeng Fu, Yue Tao, Weihui Xu, Kai Pan, Shouwei Liu

**Affiliations:** ^1^Department of Horticulture, Northeast Agricultural UniversityHarbin, China; ^2^Department of Agronomy, Heilongjiang Bayi Agricultural UniversityDaqing, China; ^3^Heilongjiang Provincial Key University Laboratory of Cold Area Vegetable Biology, Northeast Agricultural UniversityHarbin, China; ^4^Department of Life Science and Agroforestry, Qiqihar UniversityQiqihar, China

**Keywords:** tomato, potato onion, intercropping, phosphobacteria, alkaline phosphatase gene, phosphorus-rich soil

## Abstract

**Background and Aims:** In China, excessive fertilization has resulted in phosphorus (P) accumulation in most greenhouse soils. Intercropping can improve the efficiency of nutrient utilization in crop production. In this study, pot experiments were performed to investigate the effects of intercropping with potato onion (*Allium cepa* L. var. *aggregatum* G. Don) on tomato (*Solanum lycopersicum* L.) seedlings growth and P uptake, the diversity of rhizosphere phosphobacteria and alkaline phosphatase (*ALP*) genes in phosphorus-rich soil.

**Methods:** The experiment included three treatments, namely tomato monoculture (TM), potato onion monoculture (OM), and tomato/potato onion intercropping (TI-tomato intercropping and OI-potato onion intercropping). The growth and P uptake of tomato and potato onion seedlings were evaluated. The dilution plating method was used to determine the population of phosphate-solubilizing bacteria (PSB) and phosphate-mineralizing bacteria (PMB). The genomic DNAs of PSB and PMB in the rhizosphere of tomato and potato onions were extracted and purified, and then, with the primer set of 338f /518r, the PCR amplification of partial bacterial 16S rDNA sequence was performed and sequenced to determine the diversities of PSB and PMB. After extracting the total genomic DNAs from the rhizosphere, the copy numbers and diversities of *ALP* genes were investigated using real-time PCR and PCR-DGGE, respectively.

**Results:** Intercropping with potato onion promoted the growth and P uptake of tomato seedlings, but inhibited those of potato onion. After 37 days of transplanting, compared to the rhizosphere of TM, the soil pH increased, while the electrolytic conductivity and Olsen P content decreased (*p* < 0.05) in the rhizosphere of TI. The populations and diversities of PSB, PMB, and *ALP* genes increased significantly in the rhizosphere of TI, compared to the rhizosphere of TM.

**Conclusion:** The results indicated that intercropping with potato onion promoted the growth and P uptake of tomato in phosphorus-rich soil and affected the community structure and function of phosphobacteria in tomato rhizosphere. Intercropping with potato onion also improved soil quality by lowering levels of soil acidification and salinization.

## Introduction

Phosphorus (P) is an important mineral nutrient for plant growth. Soil P content in the greenhouse is far beyond the suitable amount needed by vegetables because of excessive chemical fertilizer application (Vanderzee, 2001; Chen et al., 2004). The Olsen P concentration in surface soil (0–20 cm) was over 90 mg·kg^−1^ (optimized Olsen P concentration) in more than 70% of the greenhouses in China. Excessive P fertilizer would not only reduce yields but also cause soil P accumulation (Zhang et al., 2010). Excessive fertilizer application increased soil electrolytic conductivity (Ec) and decreased pH, resulting in secondary salinization and degeneration of soils (Shi et al., 2009), which, in turn, would deteriorate soil nutrient availability, plant growth, and uptake ability (He, 2004; Ghehsareh and Samadi, 2012).

Intercropping could improve soil quality (Li et al., 1999) and increase P availability in the rhizosphere of intercropped plant species (Li et al., 2007; Hinsinger et al., 2011), thereby enhancing soil resource utilization (Li et al., 2001; Zhang and Li, 2003; Javanmard et al., 2009), and increasing crop productivity (Li et al., 1999). Intercropping could also modify the dominant microbial species and microbial communities of the soils (Song et al., 2007; He et al., 2013). Previous studies demonstrated that many microbes could provide available P to plants from inorganic and organic pools via solubilizing indissolvable inorganic phosphorus and mineralizing organophosphorus, such as phosphate-solubilizing bacteria (PSB) and phosphate-mineralizing bacteria (PMB; Khan et al., 2009; Liu et al., 2011). For example, *Pseudomonas* and *Bacillus* possessed the ability of solubilizing and mineralizing phosphate (Wang et al., 2007). *Bacillus* sp. could stimulate plant growth through increasing the uptake of N, P, K, and Fe (Biswas et al., 2000). Since PSB and PMB could increase P availability of soil, it is significant to study the roles of them in intercropping. However, little information is known about how intercropping influences the communities composition of PSB and PMB, as well as P availability in phosphorus-rich soil.

Alkaline phosphatase *(ALP)* gene was a kind of functional gene responsible for the mineralization of organic monophosphatase esters in soil, and played an important role in P solubilization (Aono et al., 2004; Browne et al., 2009; Nannipieri et al., 2011). Different *ALP* gene-harboring bacteria released different amounts and activities of ALP, and the structure of *ALP* gene-harboring bacterial communities might be crucial in determining the total soil ALP activity (Sakurai et al., 2008). Studies on examining the changes of *ALP* gene diversity could provide important information on the genetic potential of the soil bacterial community and its impact on P turnover. *ALP* gene-harboring bacterial communities could be affected by some factors such as fertilization (Chhabra et al., 2013), soil pH (Ragot et al., 2015), and organic matter (Sakurai et al., 2008). However, little information is known about the effects of intercropping on the changes of *ALP* gene diversity and their relation to P availability.

Tomato (*Solanum lycopersicum* L.) is a kind of vegetable crop commonly grown in facility. Excessive fertilizer application and continuous monocropping of tomato has resulted in soil acidification and salinization, hence decreasing tomato yield and fruit quality (Liu et al., 2014). Intercropping with some companion plants could increase tomato quality, suppress nematodes, and improve soil environment without decreasing tomato yield (Liu et al., 2014; Tringovska et al., 2015). Potato onion (*Allium cepa* L. var. *aggregatum* G. Don) is planted in northern regions of China. Our previous studies have found that intercropping with potato onion increased the yield of cucumber and tomato, and improved soil quality by changing the soil enzyme activities and microbial communities (Zhou et al., 2011; Wu et al., 2013). The aims of this study are to evaluate the effects of tomato/potato onion intercropping on the growth and P uptake of tomato and potato onion and investigate the changes in the structure and composition of PSB and PMB in the rhizosphere of tomato and potato onion, with the changes of *ALP* gene copy numbers and community structure of *ALP* gene-harboring bacteria in the rhizosphere of tomato and potato onion determined.

## Materials and methods

### Greenhouse experiment

This study was performed in a greenhouse located in the Experimental Center of Northeast Agricultural University in Harbin, China (45°41′N, 126°37′E) from April to July in 2013. Tomato cultivar “Dongnong708” and potato onion cultivar “Wuchang” were used in this study. Tomato seedlings with four leaves and potato onion bulbs were simultaneously planted in pots (25 cm, diameter; 18 cm, height). The soil used in the pot experiments was collected from the upper soil layer (0–15 cm) of a greenhouse in which tomato had been cultivated continuously for 8 years. The soil contained 25.20 g·kg^−1^ organic matter, 91.00 mg·kg^−1^ available N (nitrate and ammonium), 243.43 mg·kg^−1^ Olsen-P, and 323.30 mg·kg^−1^ available K; its Ec was 1.5 ms·cm^−1^ and pH was 6.61 (1:5, soil: water). About 0.5 kg of decomposed swine manure (15% organic matter, 0.5% N, 0.5% P, and 0.4% K) and 7 g of compound fertilizer (45% available nutrients, 12% N, 15% P, and 18% K) were also added to each pot as basal fertilizer.

Weeds were removed manually. Irrigation was performed twice weekly with untreated groundwater. The water content of the soil was not controlled rigorously, but frequent irrigation ensured that plants did not experience drought stress. Moreover, no standing water was left in the pots throughout the growing season (Zhou et al., 2014). The experiment included three treatments: tomato monoculture (TM), tomato/potato onion intercropping (TI-tomato intercropping and OI-potato onion intercropping), and potato onion monoculture (OM). To manage the water and fertilizer accurately, pot experiments were conducted to simulate a field trial. One tomato per pot was used in each pot in TM cultivation. In tomato/potato onion intercropping, one tomato seedling was planted into 1 pot with 4 potato onions. Four potato onions were planted into 1 pot in OM cultivation. The experimental design was a randomized complete block design with three replicates. Three treatments were performed in each block, and 40 pots were included in each treatment. That makes 120 pots in total per block. Then, there were 3 blocks, making a total of 360 pots.

### Plant sampling and analysis

Plants were harvested 23, 30, and 37 days after transplanting (DAT). Clean tomato and potato onion seedlings were dried separately in an oven at 75°C for 72 h to measure the plant dry weight (DW). To analyze the morphological parameters of tomato seedling roots, the roots of tomato seedlings were harvested carefully at 37 DAT and scanned by a root analyzer (LA-S2400; Xu et al., 2015). The total P content in shoots of tomato seedling was measured according to the method of Bao (2000); briefly, about 0.1 g of dry tomato shoot was digested in 6 mL of H_2_SO_4_−H_2_O_2_ mixture (98% sulfuric acid, hydrogen peroxide (300 g·L^−1^); volume ratio = 5:1) until clarity. P content was expressed as mg P·g^−1^ DW. P uptake was quantified per plant, and the results of shoot P uptake are presented on a DW basis (Bao, 2000). The formula of P uptake is as follows: P uptake (mg) = [P concentration in shoot (mg·g^−1^) × shoot dry weight (g)] (Betencourt et al., 2012), and P uptake was expressed as mg P·plant^−1^ DW.

### Rhizosphere soil sampling

The rhizosphere samples of tomato and potato onion in different treatments were collected at 23, 30, and 37 DAT, according to the methods described by Wang et al. (2009). Briefly, the roots of tomato and potato onion were carefully harvested from soils and shaken gently to remove the bulk soils. The soils adhering to the roots, which were collected using a brush, were treated as rhizosphere soil samples. To eliminate errors caused by environment and individual variation, the soils from 10 pots of per treatment in per block were pooled together as one biological replicate. There were total 3 blocks, indicating 3 biological replicates (*n* = 3). The rhizosphere soils of tomato and potato onion in the intercropping were collected separately as TI and OI, respectively. The mixed rhizosphere soils were sieved through a 2-mm mesh sieve, and a part of these soils was air-dried for the measurement of chemical properties. The remaining parts of the soils were stored in 4°C and −80°C for the analysis of enzyme activity and DNA extraction, respectively (Zhou and Wu, 2012).

### Soil chemical properties analysis

The pH and Ec of the rhizosphere soil were determined by a pH meter (FE20, Shanghai, China) and conductivity meter (FE30, Shanghai, China), respectively. Soil Olsen P content was determined according to the method described by Olsen et al. (1954).

### Isolation and identification of PSB and PMB in rhizosphere soil

Soil sample (5 g) was homogenized with 45 mL of sterilized water in an Erlenmeyer flask. The homogenate was stirred at 180 rpm for 30 min. Afterward, the homogenate was serially diluted to 10^−5^ g·mL^−1^ concentration. An aliquot of 100 μL of homogenate was evenly spreaded on the nutrient agar plate. The plates were incubated at 30 ± 1°C for 96 h. The number of colonies was expressed as log CFU·g^−1^ (Hameeda et al., 2008). The agar medium composition for PSB and for PMB was prepared as described by Liu et al. (2011). Each treatment was composed of three soil samples, and each soil sample has six agar plates (replicates); thus, each treatment had a total of 18 agar plates.

After 4 days of incubation, the number of colonies on each agar plate was counted. In each treatment, 6 agar plates were randomly selected from 18 plates, and all colonies on each selected agar plate were transferred to LB medium liquid medium. After 3 days of incubation (180 rpm, 25°C), bacterial genomic DNA was extracted and purified according to the method of Sambrook and Russell (2001), and then amplified via PCR with the primers pair of 338f (5′-CCTACGGGAGGCAGCAG-3′)/518r (5′-ATTACCGCGGCTGCTGG-3′; Muyzer et al., 1993), whose amplification products were partial bacterial 16S rDNA. Each reaction mixture (20 μL) contained 8 μL of ddH_2_O, 10 ng template DNA (1 μL), 0.5 μM × 2 primer (1 μL each), and TIANGEN TaqPCR MasterMix (2×, 9 μL). PCR was performed as follows: one cycle of 5 min at 94°C, followed by 30 cycles of 30 s at 94°C, 30 s at 59°C, and 40 s at 72°C; and one cycle of 10 min at 72°C. The amplification products were sequenced in BGI Tech (Shenzhen, China). The sequences of PSB or PMB were identified by nucleotide blast using GenBank database. The size of the amplified sequences were only about 12% of the full length 16S rDNA gene with the primer pair of 338f/518r, thereafter, the accession with the highest query cover, the maximum identity (sequence-identity≥97%) and minimum E value was identified as the target bacteria. For phylogenetic analyses, all the sequences were edited and trimmed to achieve the same final length of 168 bp. Consensus phylogenetic trees were constructed using Clustalx software (version 1.83) and MEGA4.0.

### Real-time PCR analysis of *ALP* gene-harboring bacterial

The abundances of *ALP* gene-harboring bacterial community were estimated by measuring the bacterial *ALP* gene abundance. Total DNA was extracted from soils using a kit from OMEGA Bio-TEK (USA). SYBR Green real-time PCR was conducted in IQ5 real-time PCR system (Bio-Rad USA) with primer ALPS-F730 (5′-CAGTGGGACGACCACGAGGT-3′)/ALPS-R1101 (5′-GAGGCCGATCGGCAT GTCG-3′; Sakurai et al., 2008). Each reaction mixture (20 μL) contained ddH_2_O (8 μL), 10 ng template DNA (1 μL), 0.5 μM × 2 primer (1 μL each), and SYBR®Premix Ex TaqTM (2 ×) (9 μL). The PCR condition was 94°C for 5 min; followed by 30 cycles of 94°C for 40 s, 59°C for 45 s, 72°C for 50 s; and a final elongation at 72°C for 10 min. Sterile water was used as negative control, and all the samples were performed in triplicate. In all cases, no inhibition was detected. Product specificity was confirmed by melting curve analysis and agarose gel electrophoresis. The *ALP* gene-harboring bacterial community was calculated through a standard curve, which was created by 10-fold dilution series of plasmids (the plasmid dilution series to the initial copy number of plasmids was from 3.22 × 10^−2^ ng·μL^−1^ to 3.22 × 10^−7^ ng·μL^−1^) from the soil samples containing *ALP* gene. The initial copy number of the target gene was determined by comparing each sample's threshold cycle (*Ct*) value and standard curve (Zhou and Wu, 2012).

### PCR amplification and DGGE analysis of *ALP* gene-harboring bacterial

DNA amplification was carried out in a Bio-Rad PCR thermocycler with ALPS-F730 (5′-CAGTGGGACGACCACGAGGT-3′)/ALPS-R1101 (5′-CGCCCGC CGCGCCCCGCGCCCGTCCCGCCGCCCCCGCCCGGAGGCCGATCGGCATGTCG-3′; Sakurai et al., 2008). The PCR condition was 3 min at 94°C; followed by 35 cycles of 40 s of denaturing at 94°C, 45 s extension at 59°C, 50 s thermal insulation step at 72°C, and a final thermal insulation at 72°C for 10 min. Each reaction mixture (50 μL) contained 10 × PCR buffer (5 μL), template DNA (20 ng), dNTPs (0.2 mM), primer (1.0 μM), Mg^2+^ (3.0 mM), and Taq DNA polymerase (1 U). Identical amounts of PCR products were loaded in 10% polyacrylamide gels with denaturing gradients ranging within 45–75%. Electrophoresis was performed at a constant voltage of 80 V for 12 h in 1 TAE buffer at 60°C, with a DCode universal mutation detection system (Bio-Rad Lab, LA, USA). Gels were stained in 1:3300 (v/v) GelRed (Biotium, CA, USA) nucleic acid staining solution for 20 min. DGGE profiles were photographed with an AlphaImager HP imaging system (Alpha Innotech Corp., CA, USA) under UV light.

### Statistical analyses

Data were analyzed by one-way ANOVA and Tukey's HSD post hoc test at 5% level using the SAS 9.1.3 software. DGGE profiles and principal component analysis (PCA) were analyzed by the Quantity One software (version 4.5) and Canoco for Windows 4.5 software, respectively. The diversity of *ALP* gene-harboring bacterial communities was estimated by using the Shannon–Wiener index (*H*) and calculated as follows: *H* = −Σ(*P*i) (ln*Pi*,), where *Pi* = *ni*/*N* (*ni* is the height of peak, and *N* is the sum of all peak heights in the curve (Liu et al., 2007). Evenness index (E) was calculated from H/H_max_, where H_max_ is equal to ln(S), and S is the total number of phylotypes (Liu et al., 2007). Intercropping advantage was assessed by relative yield total (*RYT*), which is often considered an index of intercropping advantage. When the *RYT* is > 1.0, intercropping favors the growth and yield of that species. By contrast, when the *RYT* is < 1.0, then intercropping negatively affects the growth and yield of the plants grown in mixtures (Mead and Willey, 1980). The *RYT* is defined as follows: *RYT* = (*B*_*it*_*B*_*mt*_)+(*B*_*io*_*B*_*mo*_), where *B*_*it*_ and *B*_*mt*_ are the biomass of intercropped and monoculture tomato, respectively; *B*_*io*_ and *B*_*mo*_ are the biomass (DW of the sum of shoot and root) of intercropped and monoculture potato onions, respectively. The aggressivity of tomato relative to potato onions (*A*_*to*_; Willey and Rao, 1980) is defined as follows: *A*_*to*_ = *B*_*it*_/(*B*_*mt*_·*P*t)–*B*_*io*_/(*B*_*mo*_·*P*o), where *P*t and *P*o are the proportion of intercropping tomato and potato onions to the total area of the intercropping system, respectively; *P*t equal to 20%, and *P*o equal to 80%. When *A*_*to*_ is > 0, it indicates that tomato competitiveness is stronger than that of potato onions. When *A*_*to*_ is < 0, it indicates that potato onions competitiveness is stronger than that of tomato.

## Results

### Growth and P uptake

The root DW, shoot DW, and P uptake in the shoot of tomato seedling at 37 DAT were significantly higher in intercropping system than those in TM system (Table 1). However, the root DW, shoot DW, and P uptake in the shoot of potato onion seedling at 37 DAT were significantly lower than those in the OM system. The *RYT* values were 1.93, 1.76, and 1.76 at 23, 30, and 37 DAT, respectively (Table 1). The *A*_*to*_ values were 4.20, 4.56, and 5.46 at 23, 30, and 37 DAT, respectively (Table 1). P concentration was increased in tomato/potato onion intercropping system (Table 1). The root length, root surface area, root volume, root tip number, root mean diameter, and root activity of tomato seedling in the intercropping system at 37 DAT were significantly higher than those in tomato seedling monoculture (Table 2).

**Table 1:** **The shoot and root dry weights (DW), *RYT* and *A*_to_ of tomato and potato onion, at 23th, 30th and 37th day after planting**.

**Day after planting(d)**	**Crop species**	**Cropping system**	**DW (g plant^−1^)**		***RYT***	***A*_to_**	**P concentration (mg g^−1^ DW)**	**P uptake (mg pot^−1^ DW)**
			**Shoot**	**Root**	**Biomass**		**Shoot**	
23	Tomato	Monoculture	6.64 ± 0.03b	0.70 ± 0.06a			nd	nd
		Intercropping	6.94 ± 0.09a	0.83 ± 0.08a			nd	nd
	Potato	Monoculture	1.44 ± 0.05c	0.14 ± 0.01b	1.93	4.20	nd	nd
	Onion	Intercropping	1.23 ± 0.03d	0.15 ± 0.01b			nd	nd
30	Tomato	Monoculture	11.53 ± 0.66a	1.44 ± 0.12a			nd	nd
		Intercropping	12.39 ± 0.73a	1.63 ± 0.14a			nd	nd
	Potato	Monoculture	1.72 ± 0.10b	0.29 ± 0.02b	1.76	4.56	nd	nd
	Onion	Intercropping	1.34 ± 0.18c	0.22 ± 0.04b			nd	nd
37	Tomato	Monoculture	14.69 ± 0.63b	1.80 ± 0.08b			4.84 ± 0.09 d	79.87 ± 1.83b
		Intercropping	17.71 ± 0.54a	2.49 ± 0.03a			5.46 ± 0.37 c	110.22 ± 9.13a
	Potato	Monoculture	2.34 ± 0.12b	0.42 ± 0.01b	1.76	5.46	6.00 ± 0.15b	57.42 ± 1.73c
	Onion	Intercropping	1.24 ± 0.12c	0.32 ± 0.01c			6.58 ± 0.11a	33.32 ± 0.70d

P uptake and P concentration of tomato and potato onion grown as monocrops and intercrops at 37th day. Different small letters on the same column indicate significant differences at a level of p < 0.05. nd–not determined. n = 3 representing 3 biological replicates.

**Table 2:** **Effect of intercropping with potato onion on root length, root surface area, root volume, root tip number, root mean diameter and root dry weight of tomato after 37 days planting**.

**Treatment**	**Root length (m crop^−1^)**	**Root surface area (cm^2^ crop^−1^)**	**Root volume (cm^3^crop^−1^)**	**Root tip number (number crop^−1^)**	**Root mean diameter(mm)**	**Root dry weight (g crop^−1^)**
Monoculture	53.74 ± 1.72b	841.44 ± 20.44b	34.94 ± 0.76b	4961.67 ± 130.43b	0.86 ± 0.01b	1.80 ± 0.13b
Intercropping	66.38 ± 1.23a	978.49 ± 13.74a	40.91 ± 2.88a	5244.66 ± 33.51a	0.98 ± 0.02a	2.49 ± 0.24a

Different small letters on the same column indicate significant differences at a level of p < 0.05. n = 3 representing 3 biological replicates.

### Changes of olsen P, pH, and Ec value in the rhizosphere

As shown in Table 3, the Olsen P content in the rhizosphere of tomato in both monoculture and intercropping increased significantly (*p* < 0.05) on days 23 and 30; however, the decrease of Olsen P content was observed at 37 DAT. The Olsen P content in the rhizosphere of intercropped tomato was higher than that in monocultured tomato at 23 and 30 DAT, but it was lower in the intercropping system at 37 DAT compared with TM. Interestingly, the Olsen P content in the rhizosphere of potato onion was not significantly different between monocropping and intercropping. The pH of rhizosphere soil from intercropped tomato was lower at 23 and 30 DAT, but it was higher than that of rhizosphere soil from monocultured tomato at 37 DAT. The Ec values in the rhizosphere of intercropped plants (tomato and potato onion) were higher than those of rhizosphere soil from monocultured plants at 23 and 30 DAT, but it was lower than that of rhizosphere soil from monocultured tomato at 37 DAT.

**Table 3:** **The Olsen P, pH,Ec value, PSB and PMB abundance, *Alp* gene copy number of bacteria in tomato and potato onion rhizosphere soil at 23th, 30th, 37th day after planting**.

**Day after planting(d)**	**Crop species**	**Cropping system**	**Olsen P (mg kg^−1^)**	**pH**	**Ec value (ms cm^−1^)**	**Abundance PSB (× 10 ^5^ cfu g^−1^soil)**	**Abundance PMB (× 10^5^ cfu g^−1^soil)**	***Alp* gene copy number (10^7^ copies g^−1^soil)**
23	Tomato	Monoculture	244.37 ± 3.46c	6.08 ± 0.04a	2.63 ± 0.01a	1.24 ± 0.06b	0.89 ± 0.05b	12.81 ± 0.57b
		Intercropping	260.96 ± 4.47b	6.01 ± 0.05a	2.86 ± 0.22a	1.42 ± 0.05a	1.06 ± 0.05a	16.78 ± 1.78a
	Potato	Monoculture	292.94 ± 4.52a	5.96 ± 0.01a	2.18 ± 0.02b	1.18 ± 0.04bc	0.69 ± 0.06c	17.36 ± 0.69a
	Onion	Intercropping	283.51 ± 6.82a	5.94 ± 0.05a	2.37 ± 0.17b	1.01 ± 0.16c	0.86 ± 0.11bc	13.70 ± 1.21b
30	Tomato	Monoculture	275.57 ± 4.39c	6.23 ± 0.04a	2.23 ± 0.07b	0.82 ± 0.06ab	0.74 ± 0.06b	19.09 ± 1.33c
		Intercropping	290.55 ± 5.49b	6.09 ± 0.04b	2.63 ± 0.05a	0.93 ± 0.05a	0.87 ± 0.07b	17.44 ± 0.97c
	Potato	Monoculture	309.28 ± 2.42a	6.09 ± 0.06b	2.01 ± 0.02b	0.84 ± 0.02ab	0.92 ± 0.06a	25.81 ± 2.83b
	Onion	Intercropping	308.68 ± 2.15a	6.14 ± 0.01b	2.17 ± 0.09b	0.75 ± 0.10b	0.91 ± 0.02a	33.69 ± 2.92a
37	Tomato	Monoculture	244.67 ± 8.50a	6.32 ± 0.04b	1.54 ± 0.07a	0.97 ± 0.04b	0.74 ± 0.04c	13.12 ± 1.07c
		Intercropping	202.43 ± 5.95b	6.49 ± 0.06a	1.13 ± 0.10b	1.21 ± 0.05a	0.93 ± 0.05b	24.13 ± 2.62a
	Potato	Monoculture	247.47 ± 2.41a	6.19 ± 0.03c	1.78 ± 0.16a	0.99 ± 0.00b	0.97 ± 0.03b	17.54 ± 1.22b
	Onion	Intercropping	241.51 ± 2.49a	6.37 ± 0.02b	1.12 ± 0.10b	1.01 ± 0.06b	1.23 ± 0.06a	24.25 ± 2.00a

Different small letters on the same column indicate significant differences at a level of p < 0.05. n = 3 representing 3 biological replicates.

### Abundance and diversity of phosphobacteria

The result showed that the abundance of PSB decreased, and the lowest abundance was detected at 30 DAT in all the treatments. The abundance of PSB in the soil of the intercropped tomato was higher than that of the monocultured tomato; the abundance of PSB in the soil of intercropped potato onion was lower than that of the monocultured potato onion (Table 3).

The PMB population decreased in soils of monocultured and intercropped tomatoes; the lowest PMB population tomato was observed at 37 and 30 DAT, respectively. On the contrary, the populations of PMB in the soil of monoculture and intercropped potato onion gradually increased with the extension of time. The highest PMB population of intercropped potato onion was found at 37 DAT. Moreover, the copy number of PMB in soil of intercropped plants was generally higher than that in soil of monoculture plants, regardless of tomato or potato onion in monoculture soil (Table 3). The PSB and PMB gene clone libraries were far from saturation, with low coverage range of 31–48% (Table 4), thereby indicating that only the most dominant bacterial phyla were detected. The evenness index of the PSB community and Shannon diversity index of PSB and PMB were all higher in the rhizosphere soil of intercropped tomato and potato onion than those in monocultured ones (Table 4).

**Table 4:** **Effect of intercropping on diversity indices for the soil PSB and PMB communities as represented by clone libraries for 37 days**.

**Species**	**Crop Species**	**Cropping System**	**No of sequences**	**No of OTUs**	**Coverage (%)**	**Shannon-Weiner (H)**	**Evenness (E)**
PSB	Tomato	Monoculture	44	31	0.41	3.25	0.85
		Intercropping	48	37	0.31	3.41	0.90
	Potato	Monoculture	48	33	0.42	3.19	0.82
	Onion	Intercropping	48	37	0.31	3.46	0.89
PMB	Tomato	Monoculture	52	39	0.40	3.53	0.89
		Intercropping	60	44	0.40	3.61	0.88
	Potato	Monoculture	62	42	0.48	3.53	0.86
	Onion	Intercropping	62	46	0.33	3.61	0.88

n = 3 representing 3 biological replicates.

**Figure 1:**
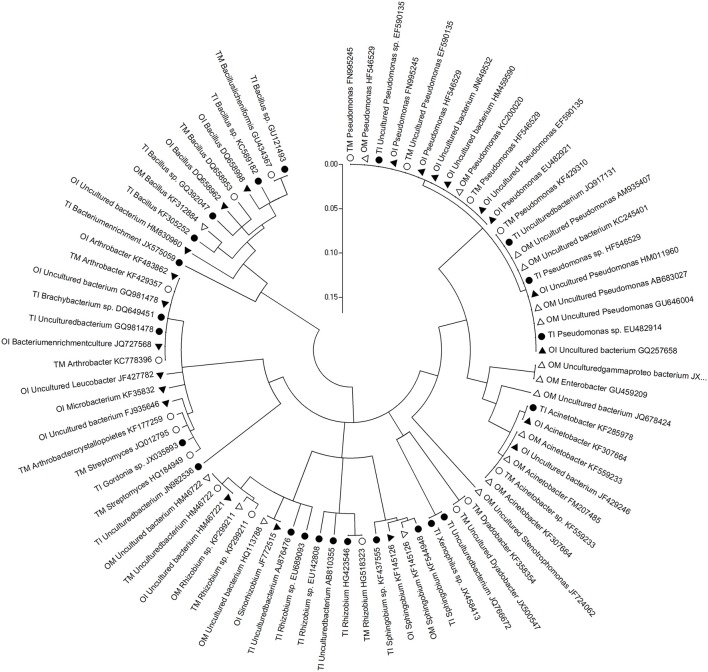
**Phylogenetic analysis based on partial bacterial 16S rDNA sequence derived from the PSB of rhizosphere soil of tomato and potato onion grown as monocrops and intercrops at 37 DAT**. Distances and clustering with the neighbor-joining method was performed by using the molecular evolutionary genetics analysis software version 4.0. TM, tomato monoculture(•); TI, tomato intercropping with potato onion(•); OM, potato onion monoculture(Δ); OI, potato onion intercropping with tomato(▴).

**Figure 2:**
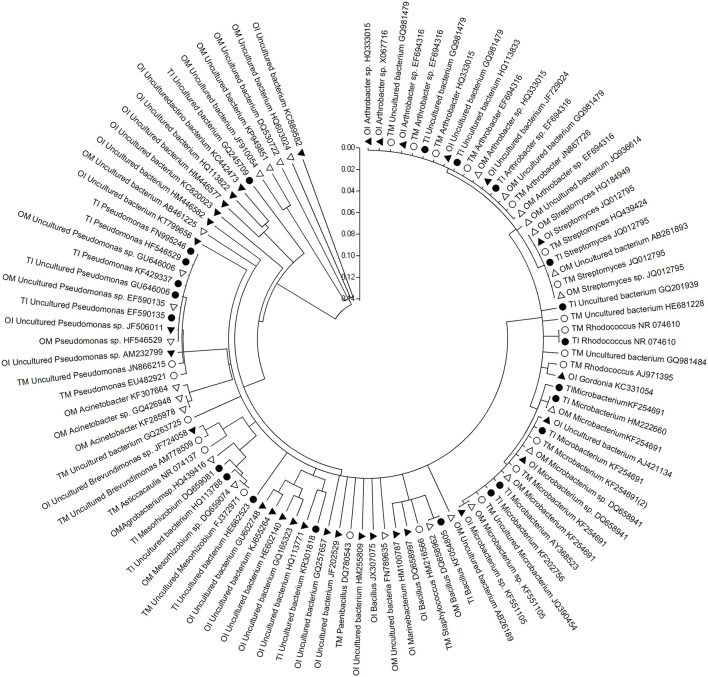
**Phylogenetic analysis based on partial bacterial 16S rDNA sequence derived from the PMB of rhizosphere soil of tomato and potato onion grown as monocrops and intercrops at 37 DAT**. Distances and clustering with the neighbor-joining method was performed by using the molecular evolutionary genetics analysis software version 4.0. TM, tomato monoculture(•); TI, tomato intercropping with potato onion(•); OM, potato onion monoculture(Δ); OI, potato onion intercropping with tomato(▴).

The differences between PSB and PMB in tomato and potato onion were significant when they were grown as monocrops or intercrops for 37 DAT (Figures 1, 2). Similarly, the community structure was different whether in intercropping or monocropping condition. Compared to monoculture, the species of phosphobacteria were increased in the rhizosphere of the intercropped plants. Sequence analysis of PSB clones cluster showed that PSB in rhizosphere soil were clustered into seven groups as follows: *Pseudomonas, Acinetobacter, Xenophilus, Sphingobium, Rhizobium, Streptomyces*, and *Bacillus*, respectively. The PSBs which had near genetic relationship with *Xenophilus* and *Rhizobium* were mainly in tomato rhizosphere soil, while those with near genetic relation to *Acinetobacter* were in potato onion rhizosphere soil. Compared to monocultured tomato, *Sphingobium* was only detected in intercropped tomato rhizosphere soil, while *Arthrobacter* only in monocultured tomato soil (Figure 1).

Sequence analysis of PMBs clones cluster showed that PMBs in rhizosphere soil were clustered into eight groups as *Arthrobacter, Streptomyces, Rhodococcus, Microbacterium, Bacillus, Mesorhizobium, Pseudomonas*, and uncultured bacteria, respectively. The PMB which had near genetic relationship with *Rhodococcus* were in tomato rhizosphere soil, and those with near genetic relation to uncultured bacteria were in potato onion rhizosphere soil. Compared to monocultured tomato, the species of uncultured bacteria closely related with *Pseudomonas* increased in the rhizosphere soil of intercropped tomato (Figure 2).

### *ALP* gene copy number and ALP gene-harboring bacteria community structure

**Figure 3:**
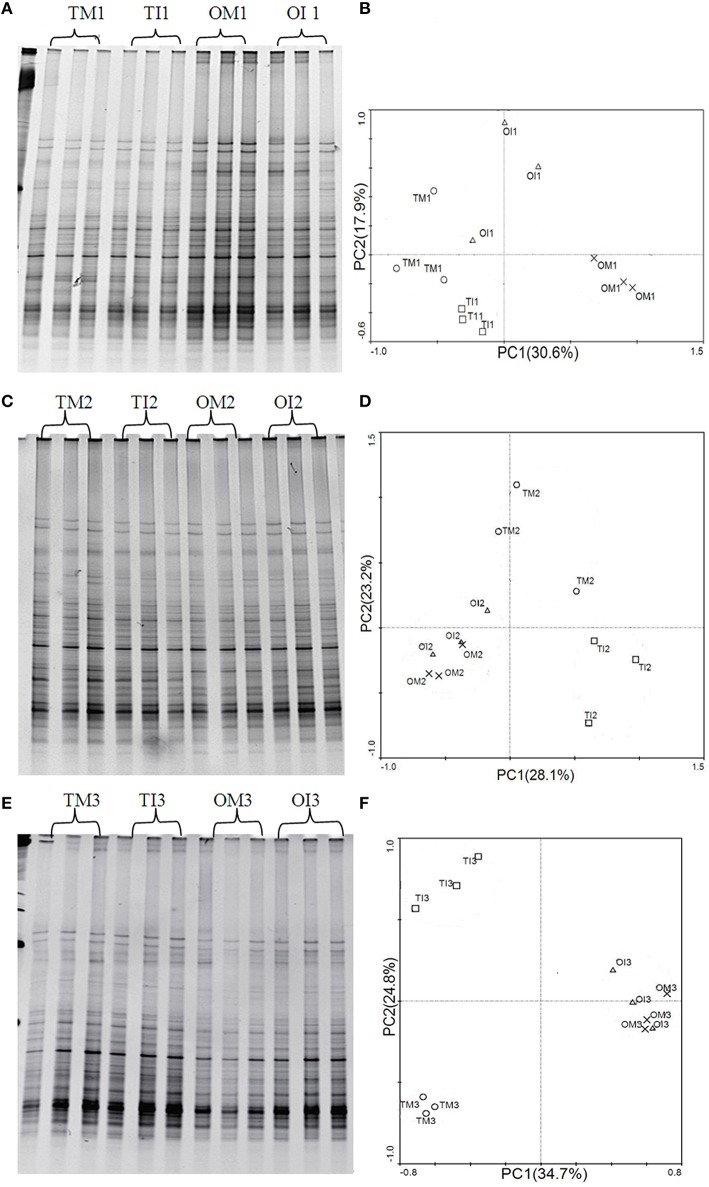
**(A)** DGGE profiles of Alkaline phosphatase (*ALP*) gene in tomato and potato onion rhizosphere soil when plants were grown as monocrops and intercrops at 23 DAT. **(B)** PCA of the *ALP* gene-harboring bacterial community based on DGGE profiles at 23 DAT. **(C)** DGGE profiles of *ALP* gene in tomato and potato onion rhizosphere soil when plants were grown as monocrops and intercrops at 30 DAT. **(D)** PCA of the *ALP* gene-harboring bacterial community based on DGGE profiles at 30 DAT. **(E)** DGGE profiles of *ALP* gene in tomato and potato onion rhizosphere soil when plants were grown as monocrops and intercrops at 37 DAT. **(F)** PCA of the *ALP* gene-harboring bacterial community based on DGGE profiles at 37 DAT. TM, tomato monoculture; TI, tomato intercropping with potato onion; OM, potato onion monoculture; OI, potato onion intercropping with tomato.

Real-time PCR assays showed that compared to monoculture, intercropping increased the abundance of *ALP* gene in the microbial community of tomato rhizosphere at 37 DAT (Table 3); similar trend was observed for potato onion (Table 3). PCR-DGGE analyses showed that *ALP* gene-harboring bacterial community changed significantly in response to the treatments; these observations indicated that the changes of *ALP* gene-harboring bacterial community structure in the rhizosphere of intercropped tomato were influenced by the extension in time (37 day; Figure 3).

PCA analysis derived from DGGE patterns showed that monoculture and intercropped samples of tomato were decentralized, whereas samples from potato onions were grouped together at 30 and 37 DAT (Figure 3). Compared to monoculture, the number of visible bands, Shannon index, and Evenness index were increased in soil samples from intercropped tomato (Table 5). These indexes were significantly decreased in soils from the potato onion at 23 DAT and tended to be stable at 37 DAT.

**Table 5:** **Number of visible bands (*S*), Shannon diversity index (*H*) and Evenness (*E*) of ALP - harboring bacteria communities when plants were grown as monocrops and intercrops for 23, 30, and 37 days**.

**Day after planting (d)**	**Crop species**	**Cropping system**	***S***	***H***	***E***
23	Tomato	Monoculture	30.33 ± 0.47b	3.25 ± 0.02b	0.82 ± 0.01b
		Intercropping	33.67 ± 1.25a	3.39 ± 0.03a	0.84 ± 0.01ab
	Potato Onion	Monoculture	35.67 ± 0.94a	3.42 ± 0.02a	0.85 ± 0.01a
		Intercroping	29.67 ± 0.47b	3.27 ± 0.05b	0.82 ± 0.01b
	P^a^		^***^	^**^	^*^
	LSD^b^		2.72	0.10	0.03
30	Tomato	Monoculture	30.67 ± 0.47b	3.26 ± 0.04b	0.83 ± 0.01b
		Ipntercropping	34.33 ± 1.24a	3.37 ± 0.03a	0.86 ± 0.01a
	Potato Onion	Monoculture	31.67 ± 0.47b	3.28 ± 0.02ab	0.84 ± 0.01b
		Intercropping	31.67 ± 0.47b	3.24 ± 0.03b	0.83 ± 0.01b
	P^a^		^**^	^*^	^*^
	LSD^b^		2.38	0.09	0.02
37	Tomato	Monoculture	25.67 ± 0.47b	3.06 ± 0.02ab	0.79 ± 0.01ab
		Intercropping	28.33 ± 0.47a	3.13 ± 0.02a	0.81 ± 0.01a
	Potato Onion	Monoculture	25.66 ± 1.25b	3.07 ± 0.04ab	0.79 ± 0.01ab
		Intercropping	26.00 ± 0.82ab	2.99 ± 0.06b	0.77 ± 0.02b
	P^a^		^*^	^*^	^*^
	LSD^b^		2.61	0.12	0.03

a P from one-way ANOVA,

***, **, * *< 0.001, 0.01, 0.05, respectively*.

b *b, Least significant difference (P = 0.05)*.

*Data are expressed as means with standard errors*.

*n = 3 representing 3 biological replicates*.

## Discussion

Intercropping could promote crop growth (Li et al., 2001; Zhou et al., 2011). In the present study, the root and shoot DW of tomato seedlings in TI were higher than those in TM, but opposite trend was obtained for potato onion seedlings (Table 1). Our results demonstrated that intercropping promoted the growth of tomato, whereas inhibited that of potato onion. These results implied that interspecific competition occurred, and tomato had greater competitive ability than potato onion when they were grown together. Our study was consistent with the aggressivity analysis described by Willey and Rao (1980); in this analysis, the *A*_*to*_ (aggressivity of tomato relative to potato onions) was > 0 (Table 1), indicating the stronger competitiveness of tomato. To evaluate the total effects of intercropping, we calculated the *RYT* which was generally considered as an index of intercropping advantage (Mead and Willey, 1980). The result showed that the *RYT* was > 1.0 (Table 1), indicating an intercropping advantage of tomato/potato onion intercropping system. These results suggested that tomato/potato onion intercropping might be an efficient strategy for tomato production.

Plant interspecies interaction could affect the growth and root morphology (de Kroon, 2007; de Kroon et al., 2012). Our study demonstrated that the morphological indices of tomato roots in intercropping system increased significantly, which was more than those in monoculture system (Table 2). The changes of the root morphology of tomato seedling, specifically the increase of the root surface area and volume, may be beneficial for nutrient uptake (Hermans et al., 2006; Cahill et al., 2010). To verify this claim, we examined the total P uptake of tomato in both intercropping and monoculture systems. The result showed that the total P uptake of tomato seedling in intercropping was significantly higher than that in TM (Table 1). This observation was consistent with the results of Zuo et al. (2003), in which intercropping with maize could increase the root length and number of lateral roots, and the changes of the root morphology might contribute to the Fe nutrition of peanut.

The increase of P uptake was partly attributed to the changes of tomato root morphology in intercropping (Cahill et al., 2010). The increased available phosphorus (Olsen P) content in the rhizosphere of intercropped tomato may also be another reason (Cu et al., 2005). In this study, the available phosphorus in terms of Olsen P content in the rhizosphere of tomato intercropped with potato onion was higher than that of tomato monocultured at 23 and 30 DAT. This may contribute to tomato P uptake in tomato/potato onion intercropping system. The phosphobacteria may be involved in the increased available phosphorus for their function of solubilizing and mineralizing P (Khan et al., 2009; Liu et al., 2011). To confirm this, we examined the abundance of PSB and PMB. The result showed that the abundance of PSB and PMB in the soil of the intercropped tomato was higher than that of the monocultured tomato, indicating increased PSB and PMB may be one of the reasons for the increase of available phosphorus in tomato rhizosphere. However, at 37 DAT, intercropping decreased the Olsen P content in tomato rhizosphere soil (Table 3). The decrease of Olsen P content at 37 DAT may be caused by the increased P uptake of the tomato seedlings, which may result in a temporary P decrease in the rhizosphere (Nuruzzaman et al., 2006). Nevertheless, the Olsen P content in the rhizosphere of intercropped tomato was 202.43 mg·kg^−1^, which was sufficient for tomato growth. Therefore, the increase of P uptake of intercropped tomato may mainly depend on the changes of root morphology.

The growth and development of plant species need healthy soil environment. Soil microbial diversity has been regarded as an important indicator of soil quality (Kong et al., 2011). Previous studies showed that intercropping with potato onion or garlic could improve the microbial diversity of cucumber rhizosphere (Zhou et al., 2011), increase the pH, and decrease the Ec value of tomato rhizosphere (Liu et al., 2014). The same phenomenon was observed in this study, which showed that intercropping with potato onion increased the number and diversity of PSB and PMB (Tables 3, 4). In addition, at 37 DAT, intercropping decreased the soil Ec value and increased the soil pH (Table 3). These results indicated that intercropping with potato onion might improve the soil environment of tomato rhizosphere by changing the microbial community structure and lowering the levels of soil acidification and salinization.

In soil, there were abundant inorganic phosphorus and organophosphorus that could not utilized by plants. Interestingly, many microbe species, such as PSB and PMB, could solubilize and mineralize inorganic phosphorus and organophosphorus to be available phosphate forms which could be utilized by plants (Khan et al., 2009; Liu et al., 2011). In this study, many phosphobacteria species in tomato rhizosphere soil were detected (Figures 1, 2). For example, *Sphingobium*, a PSB in soil ecosystem (Hashidoko et al., 2006; Ulrich et al., 2008), was detected only in the intercropped tomato rhizosphere (Figure 1). Furthermore, the Shannon diversity and Evenness indexes of the PSB community were high in the intercropping soil of tomato and potato onion (Table 4). This result signifies the increased diversity and function of phosphobacteria in the rhizosphere of tomato intercropped with potato onion under this experimental condition. This is similar to the study in which intercropping increased P uptake by selective enrichment of competent species (He et al., 2013). But a limitation need to be noted that examining the diversity of PSB and PMB by plate culturing method was only suitable for the fast-growing species, hence missing the noncuturable bacteria. Therefore, non-culturing method should be employed to examine the all phosphobacteria species in further study. Attention should be paid on the sequence size of amplification products with the primer pairs of 338f /518r, which was only 12% of the full length 16S rDNA gene, so the taxonomic assignment which can be achieved by sequence comparison in public databases was limited. This may affect the result in terms of PSB and PMB diversity in this study. In addition, it has been universally acknowledged that mycorrhiza can also help plant to acquire phosphorus and other nutrients (Smith et al., 2011), deserving to be further study in tomato/potato onion intercropping.

It has been universally reported that intercropping could alter bacterial communities (Zhou et al., 2011; He et al., 2013), and the changes in microbial community structure could be correlated with the changes in certain functions (Kandeler et al., 2002; Avrahami et al., 2003). Importantly, the *ALP* gene-harboring bacterial community structure was in part significantly correlated with ALP activity, hence affecting the organophosphorus solubilization (Sakurai et al., 2008). In this study, many *ALP* genes were detected in the DGGE profile of each sample (Figures 3A,C,E), indicating a large diversity of *ALP* gene-harboring bacteria. The PCA of the *ALP* gene DGGE profiles indicated that community structures were affected by plant species or cropping system (Figures 3B,D,F). In addition, intercropping with potato onion significantly increased the quantity and diversity of bacterial community containing *ALP* gene in tomato rhizosphere (Tables 3, 5), and these results confirmed a fact that intercropping with potato onion changed the community composition of *ALP* gene-harboring bacterial (Figure 3F). Therefore, intercropping would increase the potential ability of solubilizing and mineralizing inorganic phosphorus and organophosphorus. This may be one of the reasons for phosphorus nutrition improvement of tomato in tomato/potato onion companion cropping system.

## Conclusion

Intercropping with potato onion promoted the growth of tomato but inhibited that of potato onion. Changes of the root morphology of tomato seedling in intercropping, specifically the increase of root surface area and volume, were beneficial for P uptake. Additionally, intercropping (37 DAT) decreased the soil Ec value, and increased the soil pH, phosphobacteria diversity and function in the rhizosphere of tomato intercropped with potato onion. Intercropping with potato onion may be an effective strategy in tomato production through improving soil environment and phosphorus nutrition.

## Author contributions

All the authors declared that everyone contributed adequately to all the procedures of the experiment and manuscript writing. XW made the main contributions to the design and performance of the work; acquisition, analysis, and interpretation of data for the study; XW also drafted the manuscript and made critical revision for the whole content. FW designed the work and revised the manuscript critically for the main content. XZ, XF, YT, and WX made contributions to the acquisition and analysis of data; they also revised the manuscript critically for important intellectual content. KP and SL made contributions to the interpretation of data for the work and revised the manuscript. All authors approved the final version for publication and agreed to be accountable for all aspects of the work to ensure that questions related to the accuracy or integrity of any part of the work are appropriately investigated and resolved.

### Conflict of interest statement

The authors declare that the research was conducted in the absence of any commercial or financial relationships that could be construed as a potential conflict of interest.
